# Revealing Glycoproteins in the Secretome of MCF-7 Human Breast Cancer Cells

**DOI:** 10.1155/2015/453289

**Published:** 2015-06-17

**Authors:** Aik-Aun Tan, Wai-Mei Phang, Subash C. B. Gopinath, Onn H. Hashim, Lik Voon Kiew, Yeng Chen

**Affiliations:** ^1^Institute for Research in Molecular Medicine (INFORMM), Universiti Sains Malaysia, 11800 Penang, Malaysia; ^2^Department of Oral Biology & Biomedical Sciences, Faculty of Dentistry, University of Malaya, 50603 Kuala Lumpur, Malaysia; ^3^Institute of Nano Electronic Engineering (INEE) and School of Bioprocess Engineering, Universiti Malaysia Perlis, 01000 Kangar, Perlis, Malaysia; ^4^Department of Molecular Medicine, Faculty of Medicine, University of Malaya, 50603 Kuala Lumpur, Malaysia; ^5^Department of Pharmacology, Faculty of Medicine, University of Malaya, 50603 Kuala Lumpur, Malaysia; ^6^Oral Cancer Research and Coordinating Centre, Faculty of Dentistry, University of Malaya, 50603 Kuala Lumpur, Malaysia

## Abstract

Breast cancer is one of the major issues in the field of oncology, reported with a higher prevalence rate in women worldwide. In attempt to reveal the potential biomarkers for breast cancer, the findings of differentially glycosylated haptoglobin and osteonectin in previous study have drawn our attention towards glycoproteins of secretome from the MCF-7 cancer cell line. In the present study, further analyses were performed on the medium of MCF-7 cells by subjecting it to two-dimensional analyses followed by image analysis in contrast to the medium of human mammary epithelial cells (HMEpC) as a negative control. Carboxypeptidase A4 (CPA4), alpha-1-antitrypsin (AAT), haptoglobin (HP), and HSC70 were detected in the medium of MCF-7, while only CPA4 and osteonectin (ON) were detected in HMEpC medium. In addition, CPA4 was detected as upregulated in the MCF-7 medium. Further analysis by lectin showed that CPA4, AAT, HP, and HSC70 were secreted as N-glycan in the medium of MCF-7, with HP also showing differentially N-glycosylated isoforms. For the HMEpC, only CPA4 was detected as N-glycan. No O-glycan was detected in the medium of HMEpC but MCF-7 expressed O-glycosylated CPA4 and HSC70. All these revealed that glycoproteins could be used as glycan-based biomarkers for the prognosis of breast cancer.

## 1. Introduction

Breast cancer occurs predominantly in the female population. A few cases of breast cancer were reported in males, increasing the deaths reported worldwide. Breast cancer is a type of carcinoma formed in milk ducts and glands. If untreated, the cancer tissues will grow abnormally and spread to surrounding tissues. Different causes have been proposed for the development of breast cancer [1, 2]. Of all the factors involved, older women and those with a family history of breast cancer have a higher chance of being affected by breast cancer. Apart from these factors, the involvement of noncoding RNA and micro-RNA in cancer progression has also been reported [3–5]. Gopinath et al. [4] have revealed that noncoding RNA resides in the vault particles of cancer cells responsible for multidrug resistance. Meanwhile, Isobe et al. [5] demonstrated the regulation of tumorigenicity in breast cancer stem cells by the miR-142 micro-RNA through the canonical WNT signaling pathway. For different reasons, breast cancer has accounted for over 25% of all cancers diagnosed and causes death in a significant proportion of cases [6–8]. Based on the statistical reports of the American Cancer Society, estimated 231,840 new invasive breast cancer cases are expected to be diagnosed among the female population in the US in 2015 and it is estimated that 40,290 deaths from the disease will be reported in the same period.

The higher incidence rate for breast cancer is mainly due to a failure to detect it in the early stages. Breast tomosynthesis, 3D imaging techniques, and digital mammography are the methods currently used to diagnose breast cancer. In addition, several other detection systems have also been proposed [9–13]. These analyses help to some extent in the stage-specific diagnosis of breast cancer; however, these detection systems are hindered by the higher expectation of additional biomarkers expressed during the cancer developing stages [10]. The present study analyzes the excretion of proteins during the growth of MCF-7 cancer cells compared to normal HMEpC cells. Excreted proteins were evaluated with the assistance of proteomics using two-dimensional analyses followed by imaging analyses. Moreover, the focus of this study is the analyses of glycoproteins, as these proteins are involved in the posttranslational modifications which could contribute to tumorigenesis [14, 15]. Hence, these observations might lead to the discovery of biomarkers for early diagnosis of breast cancer.

## 2. Materials and Methods

### 2.1. Cell Culture

Human breast cancer cell line MCF-7 (catalogue number HTB-22) and human mammary epithelial cell HMEpC (catalogue number 830K-05a) were purchased from ATCC and Cell Applications, respectively. MCF-7 cells were cultured in Dulbecco's modified Eagle's medium (DMEM) containing 10% fetal bovine serum (Invitrogen, CA, USA). For HMEpC, cells were cultured in Mammary Epithelial Cell Growth Medium (Cell Applications, CA, USA) as recommended by a manufacturer. Both cell lines were maintained in a humidified atmosphere of 5% CO_2_ at 37°C. The cells were kept separately and handled individually to prevent cross-contamination. Cell growth was monitored and maintained at logarithmic growth phase.

### 2.2. Sampling of Growth Medium

The cells were cultured in 75 cm^2^ flasks until 80% confluence and the used growth media were then removed. The cells were washed three times with phosphate buffered saline (PBS) (Invitrogen), pH 7.4, and incubated for another 24 h in serum-free media. Serum-free media were harvested, centrifuged at 2000 ×g to remove cell debris, and kept in −80°C until further processing. Before being subjected to two-dimensional electrophoresis (2D-E), the harvested media were concentrated 100-fold using Vivaspin concentrators (10,000 molecular weight cut-off; Sartorius) and impurities were removed with 2D Clean-Up Kit (GE Healthcare Bio-Sciences, Uppsala, Sweden).

### 2.3. Two-Dimensional Electrophoresis (2D-E) and Silver Staining

2D-E was carried out as previously described [16]. Immobilized pH gradient (IPG) strips (GE Healthcare Bio-Sciences) with length of 13 cm and immobilized pH gradients of 3–10 and 4–7 were used. The broad range pH 3–10 IPG strip was used to view the overall protein distribution of the sample, while the pH 4–7 IPG strip was used to produce a higher resolution of the protein profile. Concentrated protein samples from growth media were rehydrated with IPG strips in a rehydration buffer (8 M urea, 2 M thiourea, 20 mM dithiothreitol, 4% CHAPS, and 0.5% pharmalyte) and incubated overnight. The strips were then subjected to isoelectric focusing (IEF) using the Ettan IPGphor II IEF system (GE Healthcare Bio-Sciences). The strips were subsequently equilibrated and applied onto the 8–18% gradient gels for second dimensional separation. The SDS-PAGE was performed using the Hoefer SE 600 Ruby system (GE Healthcare Bio-Sciences). The 2D-E gels were silver stained according to Heukeshoven and Dernick [17]. For mass spectrometry, gels were silver stained as described by Shevchenko et al. [18] with modification.

### 2.4. Con A (Concanavalin A) Affinity Chromatography for N-Linked Glycosylation Analysis

Ten milliliters of harvested medium was added to 2 mL of Con A Sepharose (GE Healthcare Bio-Sciences, Uppsala, Sweden) and gently shaken overnight at 4°C. The mixture was subsequently loaded into a 0.8 × 4 cm Poly-Prep column (BioRad Laboratories, Hercules, CA, USA) and equilibrated with an equilibration buffer (20 mM Tris-HCl, 0.5 M NaCl, pH 7.4). The column was washed with 50 mL of equilibration buffer to remove unbound proteins (nonglycosylated and O-glycosylated proteins); and bound N-glycoproteins were eluted with 0.3 M methyl-*α*-D-glucopyranoside. Chromatographic process was monitored at the absorbance of 280 nm. The eluted fractions were pooled and concentrated 100-fold using Vivaspin concentrators (10,000 molecular weight cut-off; Sartorius). Concentrated eluate was further desalted using 2D Clean-Up Kit (GE Healthcare Bio-Sciences, Uppsala, Sweden) and subjected to 2D-E.

### 2.5. Western Blotting and Champedak Galactose Binding Lectin Detection for O-Linked Glycosylation Analysis

Proteins in 2D-E growth media gel were electroblotted onto a nitrocellulose membrane (0.45 *μ*m) using Multiphor II NovaBlot Kit (GE Healthcare Bio-Sciences, Uppsala, Sweden). The blotted membrane was then incubated with 5% skimmed milk in Tween TRIS-buffered saline (TTBS) for 1 hour at room temperature to block nonspecific protein binding sites. The membrane was then washed 3 times with TTBS, 15 min each. Detection of transferred O-glycosylated proteins was performed by incubation with champedak galactose binding lectin conjugated to horseradish peroxidase at a concentration of approximately 1 *μ*g/mL, overnight at 4°C. The purity and specificity of this lectin to interact with O-glycosylated proteins were described previously [19]. After the incubation, the membrane was washed twice and developed using freshly prepared 3,3′-diaminobenzidine (Dako, Glostrup, Denmark) in 50 mL of TRIS-buffered saline mixed with 50 *μ*L of H_2_O_2_. Reaction was terminated by washing the membrane twice with deionized distilled water, 5 min each. The developed membrane was air-dried and scanned with GS-710 Imaging Densitometer (Bio-Rad).

### 2.6. Image Analysis

GS-710 Imaging Densitometer (Bio-Rad) and PDQuest software (version 4.7.0, Bio-Rad) were used to capture, store, and analyze protein spots on 2D-E gels and lectin blots. PDQuest software matched the identical spots in a series of gels and normalized the gels to compensate for any variations between gels, especially those caused by varying experimental conditions. The analysis was normalized by total density in gel, which accounts for the raw quantity of each spot in a gel, divided by the total intensity value of all the pixels in the image. The normalized spot quantity was expressed as percentages of volume contributions (vol%) to facilitate the data compilation. Data was checked manually to eliminate possible error in matching pairs.

### 2.7. Statistical Analysis

All protein concentration values were presented as mean of percentage volume (% volume) ± SE. The Student's *t*-test was used to analyze the statistical differences between normal and cancer samples and to examine the correlation between the variables. A *p* value of less than 0.05 (*p* < 0.05) was considered statistically significant.

### 2.8. Protein Identification with Mass Spectrometry (MS)

The resolved protein spots of interest were excised and subjected to in-gel digestion using ProteoExtract All-in-One Trypsin Digestion Kit (Merck, USA). Digested peptides were further purified and concentrated using ZipTip C_18_ (Millipore, MA, USA). Mass spectrometric analysis was performed at the Proteomic Centre, Department of Biological Sciences, National University of Singapore. Digested peptide was mixed with 1 *μ*L of CHCA (5 mg/mL of alpha-cyano-4-hydroxycinamic acid in 0.1% trifluoroacetic acid and 50% acetonitrile in deionized distilled water) and applied to a Matrix-Assisted Laser Desorption/Ionization (MALDI) target plate. The mixture was allowed to dry under ambient temperature to ensure optimum crystal growth. The target plate with dried mixture was then inserted into a mass spectrometer for analysis. The peptide mass spectra were obtained by using the ABI 4800 Proteomics Analyzer MALDI-TOF/TOF Mass Spectrometer (Applied Biosystems, Framingham, MA, USA).

For protein identification, mass spectra obtained were searched for in the National Center for Biotechnology Information nonredundant (NCBInr) protein database using the MASCOT search engine (version 2.1; Matrix Science, London, UK). Searches were performed with fixed modification on carbamidomethylation of cysteines and variable modification of methionine oxidation. The following parameters were used in the MASCOT peptide mass fingerprint search: (i) enzyme: trypsin with one missed cleavage allowed, (ii) species:* Homo sapiens*, (iii) mass value: monoisotopic, (iv) peptide mass tolerance: ±0.1 Da, and (v) peptide charge state: 1+. The same parameters were used in the MASCOT ion search, except for peptide mass tolerance and fragment mass tolerance which were set at 100 ppm and 0.2 Da, respectively. A search score of more than 50 indicated identities or extensive homology (*p* < 0.05).

## 3. Results and Discussion

Most of the aberrantly expressed proteins are acute phase proteins, which altered their expression level in response to the inflammation associated with the development of cancer [20]. Cancer has been reported to cause unusual changes in the protein expression of cells, either by increasing or reducing the expression level or altering the posttranslational modification of the proteins. Glycosylation is a type of extensive posttranslational modification which has a significant involvement in the functional alteration of proteins and, as reported previously, the aberrant glycosylation in the cancerous cells [21–23]. Based on our preliminary study on the glycosylation of proteins from human breast cancer cells, we encountered the aberrant expression of osteonectin and haptoglobin [15]. In the present study, we have expanded this further with the similar analysis and discovered the additional candidates which undergo differential glycosylation, through comparison of the secretome from MCF-7 cells with human mammary epithelial cells (HMEpC). The MCF-7 cell line is of luminal epithelial origin and is often used as a model for estrogen receptor-positive tumors, while HMEpC are normal epithelial cells derived from normal adult mammary glands.

### 3.1. Typical Protein Profiles of MCF-7 and HMEpC Media

Silver-stained 2D-E gels of HMEpC and MCF7 growth media were scanned using GS-710 Imaging Densitometer and analyzed using PDQuest 2D gel analysis software. Interestingly, the typical protein profile of MCF-7 medium differed considerably from the representative profile of HMEpC medium, with only some spots matched between them. Comparative analysis on the gel images revealed several differentially regulated proteins in both MCF7 and HMEpC media. A total of 5 distinctive protein spots were detected, 3 spots were exclusively expressed in MCF7 cells, one was in the HMEpC cells, and the other one was found in both MCF7 and HMEpC. These protein spots were further subjected to mass spectrometric analysis for protein identification.

Identification of protein spots of interest was performed by using Proteomics Analyzer MALDI-TOF/TOF Mass Spectrometer. The mass spectra obtained were searched for in the NCBInr protein database using the MASCOT search engine. The mass spectra of all digested samples are shown in Supplementary Figure 1 (in Supplementary Material available online at http://dx.doi.org/10.1155/2015/453289). A search score of more than 50, which indicates extensive homology, was obtained for the five distinctive spots (Table 1). The proteins detected in the MCF-7 medium were identified as carboxypeptidase A4 (CPA4), alpha-1-antitrypsin (AAT), haptoglobin (HP), and HSC-70 (HSC70), whereas proteins found in the HMEpC medium were identified as carboxypeptidase A4 (CPA4) and osteonectin (ON). Protein profile analysis using PDQuest 2D gel analysis software revealed that there is a significantly higher expression of CPA4 in the MCF-7 cell line (increased by a factor of 3.41, *p* < 0.05), compared to HMEpC.

### 3.2. Posttranslational Modification Study

Analyses of growth media protein profiles were extended to include posttranslational modification studies. The protein profiles were generated by detection with Concanavalin A (Con A) and HRP-conjugated champedak galactose binding (CGB) lectin.

### 3.3. Detection of N-Glycoprotein Using Con A Chromatography

**Figure 1:**
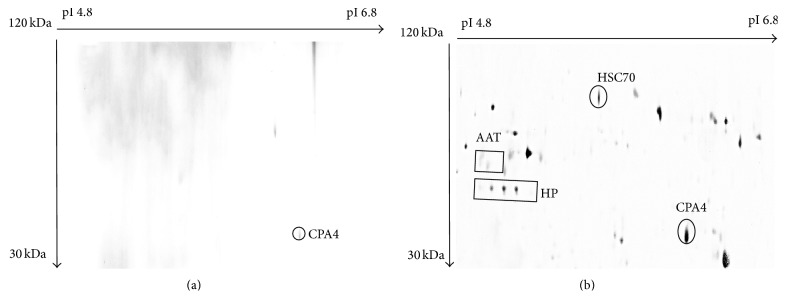
N-linked glycoproteins profile of (a) HMEpC growth medium, and (b) MCF-7 growth medium. Media of both HMEpC and MCF-7 cells were subjected to Con A chromatography and 2D-E. CPA4 was detected as N-glycoprotein in HMEpC medium, while N-glycosylated CPA4, AAT, HP, and HSC70 were detected in MCF7 medium.

Lectin Con A has high affinity towards alpha mannose, and it is thus frequently used to purify and enrich N-linked glycoproteins [24, 25]. The combination of lectin with 2D-E analysis allowed screening for possible structural aberration in oligosaccharide moieties of secreted proteins. When MCF-7 and HMEpC media were subjected to Con A coupled with 2D-E analysis, different profiles consisting of only N-glycoproteins were obtained. The glycoprotein detected in the HMEpC N-glycoprotein profile was only CPA4 (Figure 1(a)). In contrast, N-glycoproteins detected in the MCF-7 medium included CPA4, AAT, HP, and HSC70 (Figure 1(b)). As previously reported [15], image analysis on the N-glycoprotein profile of the MCF-7 medium indicated that one of the HP isoforms was absent, when compared to the total growth medium profile.

### 3.4. Detection of O-Glycoprotein Using CGB Lectin

**Figure 2:**
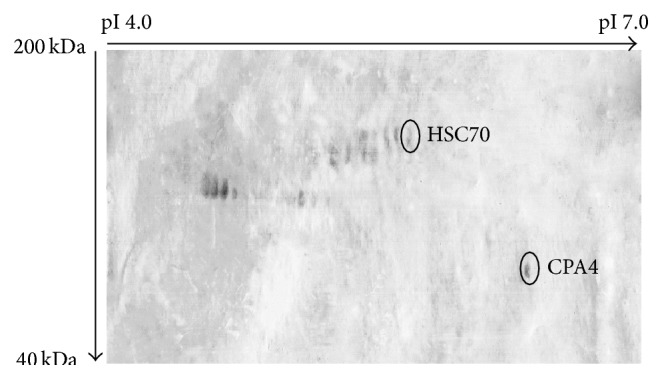
Typical representative of O-glycoprotein profile of MCF-7 growth medium. Harvested growth media were subjected to 2D-E, blotted, and then developed with HRP-conjugated CGB lectin. Protein spots of HSC70 and CPA4 were detected. No O-glycoproteins were detected in HMEpC growth medium (not shown).

For O-glycoprotein analyses, harvested growth media were subjected to 2D-E, blotted, and then developed with HRP-conjugated CGB lectin. Distinctly different profiles were obtained when 2D-E separated growth media of MCF-7 and HMEpC were exposed to HRP-conjugated CGB lectin. For HMEpC, no O-glycoproteins were detected in the growth medium. But, for MCF-7, O-glycosylated CPA4 and HSC70 were detected, as shown in Figure 2.

### 3.5. Functional Aspects of the Identified Biomarkers

**Figure 3:**
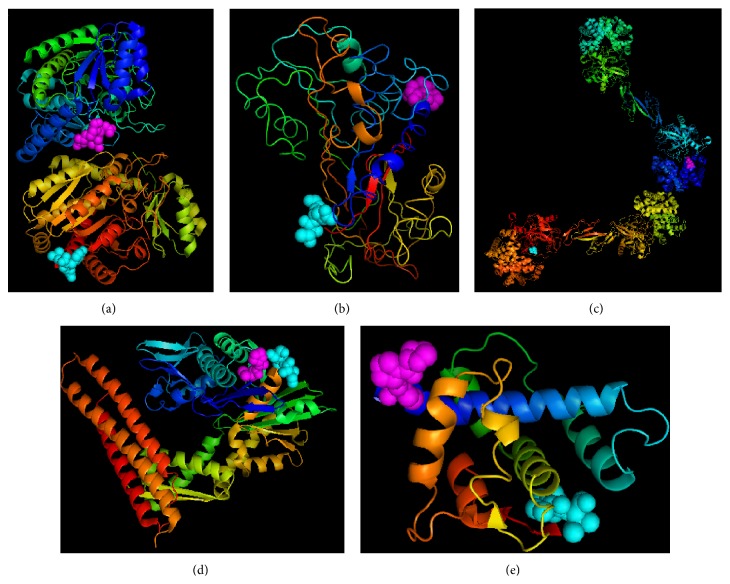
Three-dimensional representative of (a) carboxypeptidase A4 (PDB code: 2BOA), (b) alpha-1-antitrypsin (PDB code: 1KCT), (c) haptoglobin (PDB code: 4F4O), (d) HSC-70 (PDB code: 3FZF), and (e) osteonectin (PDB code: 1SRA). N and C terminals are shown with magenta and cyan colors, respectively.

With the above studies, secreted proteins from MCF-7 and HMEpC cells were comparatively analyzed with proteomic approaches involving 2D-E and glycan-binding lectin. CPA4, AAT, HP, and HSC-70 were detected in the MCF-7 medium, where AAT, HP, and HSC70 were uniquely expressed. ON was only found in HMEpC cells. Aberrantly expressed proteins in* in vitro* study of human breast cancer cell lines have different structures and functions in relation to breast cancer. The three-dimensional structures of these identified biomarkers are well studied and readily available in the protein database (Figure 3).

### 3.6. Carboxypeptidase A4 (CPA4)

CPA4 is a secreted exopeptidase that catalyzes the release of carboxyterminal amino acids. Although little is known about this enzyme, it is thought to participate in the histone hyperacetylation pathway during differentiation of prostate epithelial cancer cells [26]. Additionally, it was demonstrated that the gene for CPA4 is imprinted and may contribute to prostate cancer aggressiveness [27]. So far, no study has associated CPA4 expression with breast cancer. Our results demonstrated that CPA4 is secreted at a higher level by MCF-7 cells in comparison to HMEpC, suggesting its role in breast cancer progression. Upon analysis of N-glycoprotein profiles, CPA4 was detected in media from both MCF-7 and HMEpC cells. This finding is in accordance with a study by Pallarès et al. [28], which demonstrated that CPA4 is N-glycosylated at Asn-148 N*δ*2. However, CPA4 was also detected as an O-glycosylated protein in MCF-7 media but not in HMEpC media. Therefore, our data indicate that CPA4 is aberrantly O-glycosylated in MCF-7 cells, and this alteration may affect its function and/or structure in a manner that facilitates tumorigenesis.

### 3.7. Alpha-1-Antitrypsin (AAT)

AAT is also known as alpha-1 protease inhibitor, a 52-kDa protease inhibitor belonging to the serpin family [29] that functions as an inhibitor of caspase activation and apoptosis. Notably, studies have already identified increased levels of AAT in the serum of breast cancer patients, suggesting its association with tumor advancement [30]. In addition, Yavelow et al. [31] have also reported the expression of AAT in MCF-7 cells. Therefore, our findings confirm this correlation of AAT with breast cancer. AAT was known to be a secreted N-glycosylated protein [32], and this is compatible with our observation of N-glycosylated AAT in the medium of MCF7 cells. Changes in N-linked glycosylation during the development of cancer have been correlated with tumor progression in human breast cancer [33].

### 3.8. Haptoglobin (HP)

In parallel with our previous study [15], only the *β*-subunit of HP was studied in this investigation. Previous reports have associated the expression of HP with ovarian, breast, lung, and pancreatic cancers. Moreover, changes in oligosaccharide structures of HP variants may have contributed to tumorigenesis [34–38]. According to Chen et al. [16], the level of a secreted protein should be equivalent to its glycosylated forms. However, our findings demonstrated that HP isoforms secreted by MCF-7 cells were less N-glycosylated. Fucosylation of HP has been observed in pancreatic, breast, and ovarian cancer [38, 39]. Thus, HP may undergo differential glycosylation in associated cancer progression.

### 3.9. HSC70

HSC70 is a chaperone that facilitates proper polypeptide folding [40]. It also functions as an ATPase in the dissociation of clathrin-coated vesicles during transportation of membrane components through the cell [41]. Therefore, HSC70 is reported to be either a cytoplasmic or cell membrane-associated protein [42]. Here, we have detected extracellular HSC70 of MCF-7 cells. Although this finding could be a consequence of cell lysis or death, which is always possible in cell cultures, recent studies by Evdokimovskaya et al. [43] and Nirdé et al. [44] have supported the idea that HSC70 is actively secreted by various cell lines. So far, the mechanism and function of HSC70 secretion remain unknown. Nevertheless, our findings indicated that HSC70 is an N- and O-glycosylated protein, and this information could help to elucidate the mechanism by which it is secreted. We postulate that glycosylation could even serve as a signal for HSC70 secretion.

### 3.10. Osteonectin (ON)

ON is a secreted glycoprotein responsible for cell adhesion, proliferation, migration, and tissue remodeling [45]. Underexpression of ON has been associated with tumorigenesis in human ovarian cancer [46] and poor prognosis in breast cancer patients [47]. Our findings showed that ON was secreted in the medium of HMEpC but not in MCF-7. ON was neither detected in N-glycoprotein nor O-glycoprotein profiles of HMEpC, even though a previous study has reported that it is an N-glycoprotein [48]. These findings suggested that ON might be expressed in nonglycosylated form as N-glycosylation sequons of glycoproteins are often not glycosylated under normal circumstances [49].

## 4. Conclusions

In this study, we have identified differentially expressed and glycosylated proteins in the secretions of human breast cancer cell line MCF-7. We employed a proteomics approach by using 2D-E coupled with lectin-base analysis to identify aberrantly expressed N- and O-glycoproteins in the secretions from MCF-7 and HMEpC cells. Our analysis revealed that CPA4, AAT, HP, and HSC70 were detected in the secretion of the MCF-7 cell line and AAT, HP, and HSC70 were uniquely expressed. However, only CPA4 and ON were detected in the HMEpC medium. Image analysis revealed that CPA4 was significantly expressed in the MCF-7 medium compared to HMEpC. Further analysis by lectin showed that CPA4, AAT, HP, and HSC70 were detected as N-glycoproteins in the media of MCF-7, with HP showing differentially glycosylated isoforms. CPA4 was also detected as N-glycoprotein in the media of HMEpC. On the other hand, the MCF-7 variants of CPA4 and HSC70 were detected as O-glycoproteins, but no O-glycan was detected in HMEpC. HSC70 was detected as N- and O-glycoprotein in the lectin analysis. These findings suggest that glycol-biomarkers could be used for human breast cancer screening and molecular targets for drug development.

## Supplementary Material

Shown are the mass spectra of carboxypeptidase A4 (a), alpha-1-antitrypsin (b), haptoglobin (c), HSC-70 (d), and osteonectin (e). The protein spots were excised from the 2-DE gel, in-gel trypsin digested, ZipTip purified, and were analyzed with MALDI-TOF/TOF MS. Peaks of the tryptic peptides are shown in the spectrum.

## Figures and Tables

**Table 1:** Mass spectrometric identification of protein spots from MCF-7 and HMEpC growth media using MASCOT search engine and NCBI database.

Spot name	Protein name	Mascot accession number	Theoretical pI	Theoretical mass (Da)	Number of peaks matched	Search score
CPA4	Carboxypeptidase A4 isoform 2 preproprotein	gi∣254540196	8.49	43771	5	192
AAT	Alpha-1-antitrypsin	gi∣177827	5.42	46787	2	85
HP	Haptoglobin	gi∣223976	6.23	42344	2	70
HSC70	Chain A, crystal structure of Hsc70 BAG1 in complex with ATP	gi∣225698069	6.38	42120	3	178
ON	Osteonectin	gi∣338325	4.70	35260	1	81
