# Supplementary material for: Revealing Glycoproteins in the Secretome of MCF-7 Human Breast Cancer Cells
Source: Biomed Res Int. 2015 Jun 17;2015:453289. doi: 10.1155/2015/453289 (PMC4488092; doi:10.1155/2015/453289)
Supplement: Supplementary file 1 — Shown are the mass spectra of carboxypeptidase A4 (a), alpha-1-antitrypsin (b), haptoglobin (c), HSC-70 (d), and osteonectin (e). The protein spots were excised from the 2-DE gel, in-gel trypsin digested, ZipTip purified, and were analyzed with MALDI-TOF/TOF MS. Peaks of the tryptic peptides are shown in the spectrum. [file 453289.f1.pptx]

## Slide 1
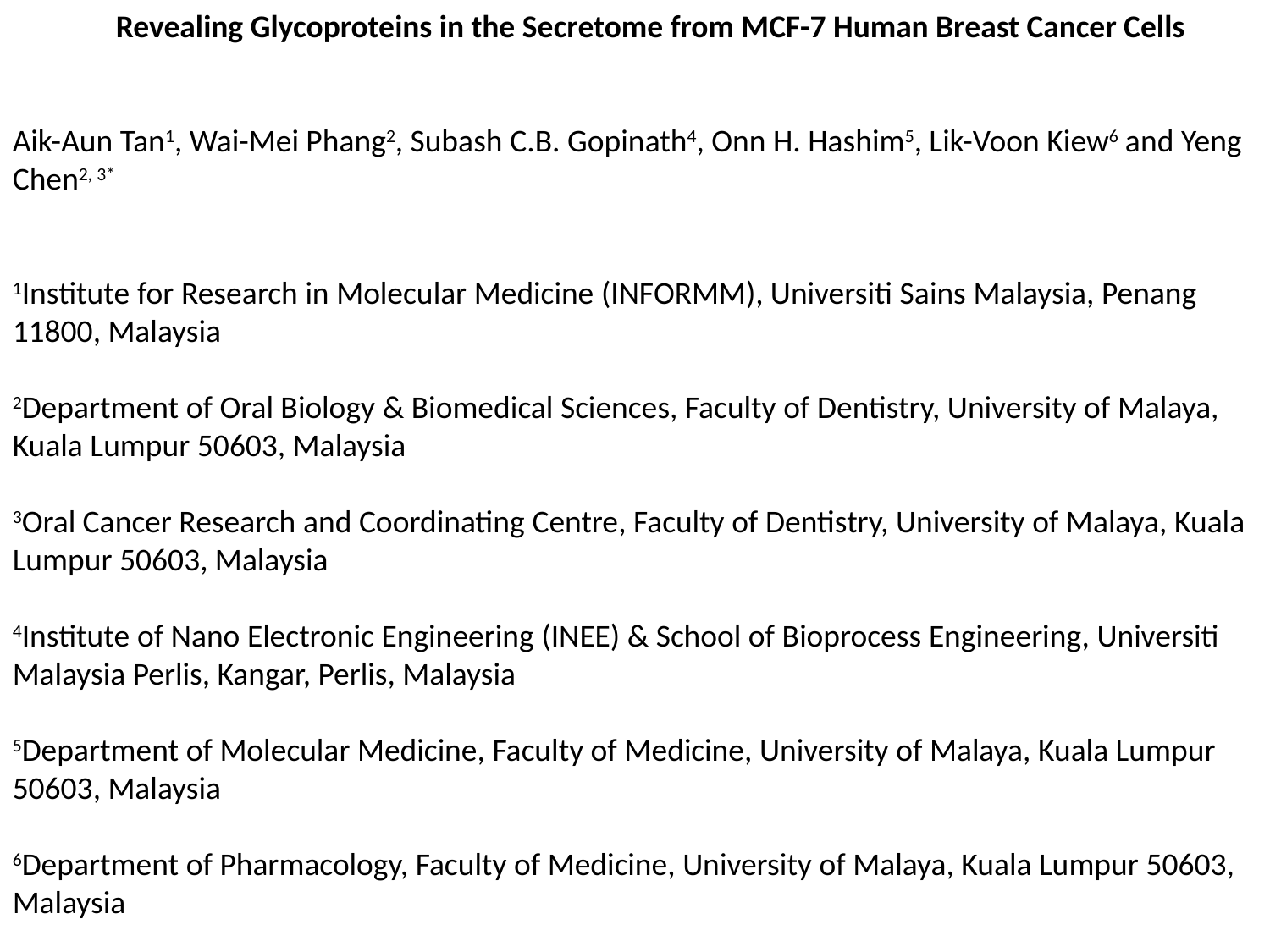

Revealing Glycoproteins in the Secretome from MCF-7 Human Breast Cancer Cells
Aik-Aun Tan1, Wai-Mei Phang2, Subash C.B. Gopinath4, Onn H. Hashim5, Lik-Voon Kiew6 and Yeng Chen2, 3*
1Institute for Research in Molecular Medicine (INFORMM), Universiti Sains Malaysia, Penang 11800, Malaysia
2Department of Oral Biology & Biomedical Sciences, Faculty of Dentistry, University of Malaya, Kuala Lumpur 50603, Malaysia
3Oral Cancer Research and Coordinating Centre, Faculty of Dentistry, University of Malaya, Kuala Lumpur 50603, Malaysia
4Institute of Nano Electronic Engineering (INEE) & School of Bioprocess Engineering, Universiti Malaysia Perlis, Kangar, Perlis, Malaysia
5Department of Molecular Medicine, Faculty of Medicine, University of Malaya, Kuala Lumpur 50603, Malaysia
6Department of Pharmacology, Faculty of Medicine, University of Malaya, Kuala Lumpur 50603, Malaysia

## Slide 2
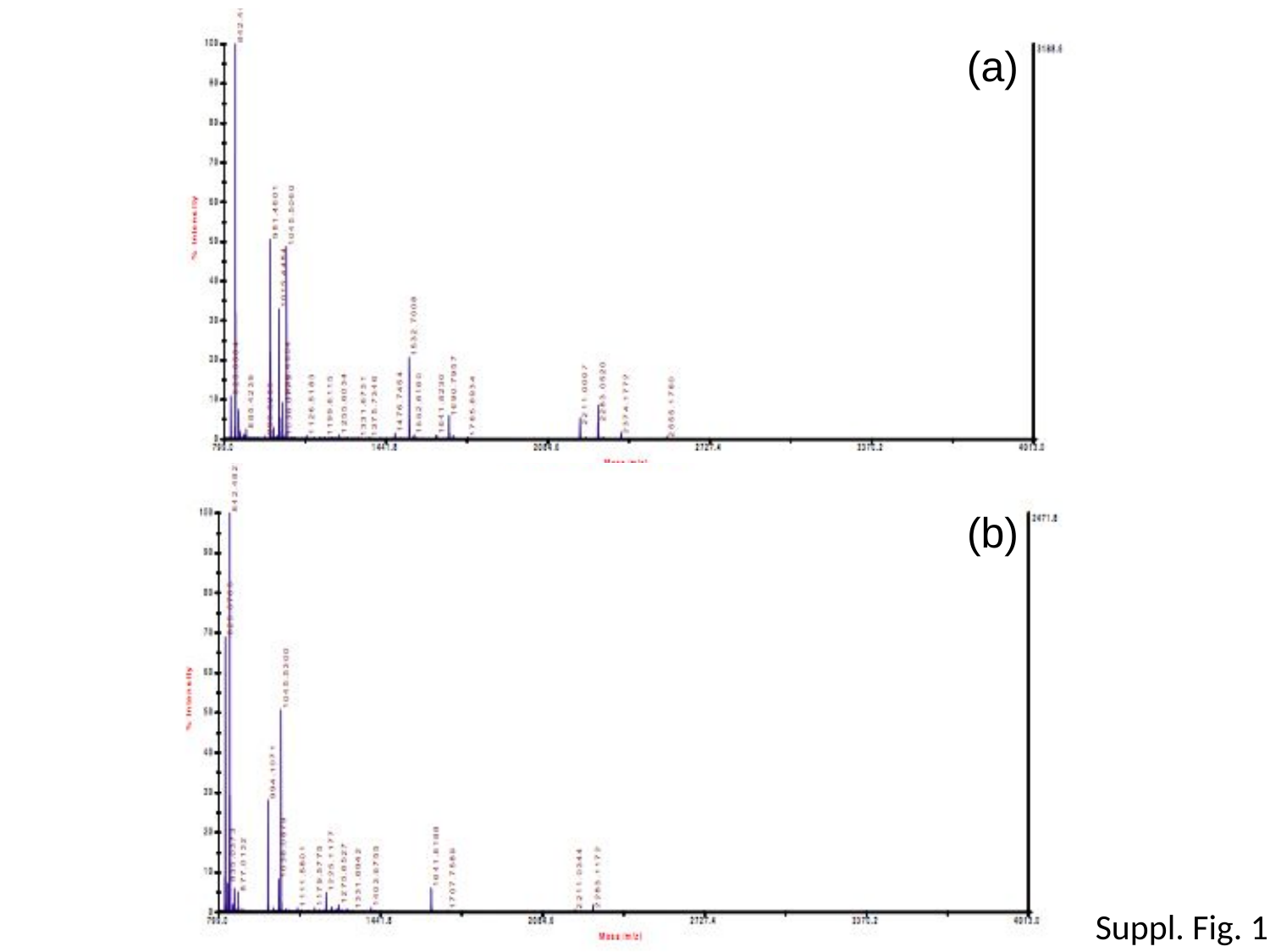

(a)
(b)
Suppl. Fig. 1

## Slide 3
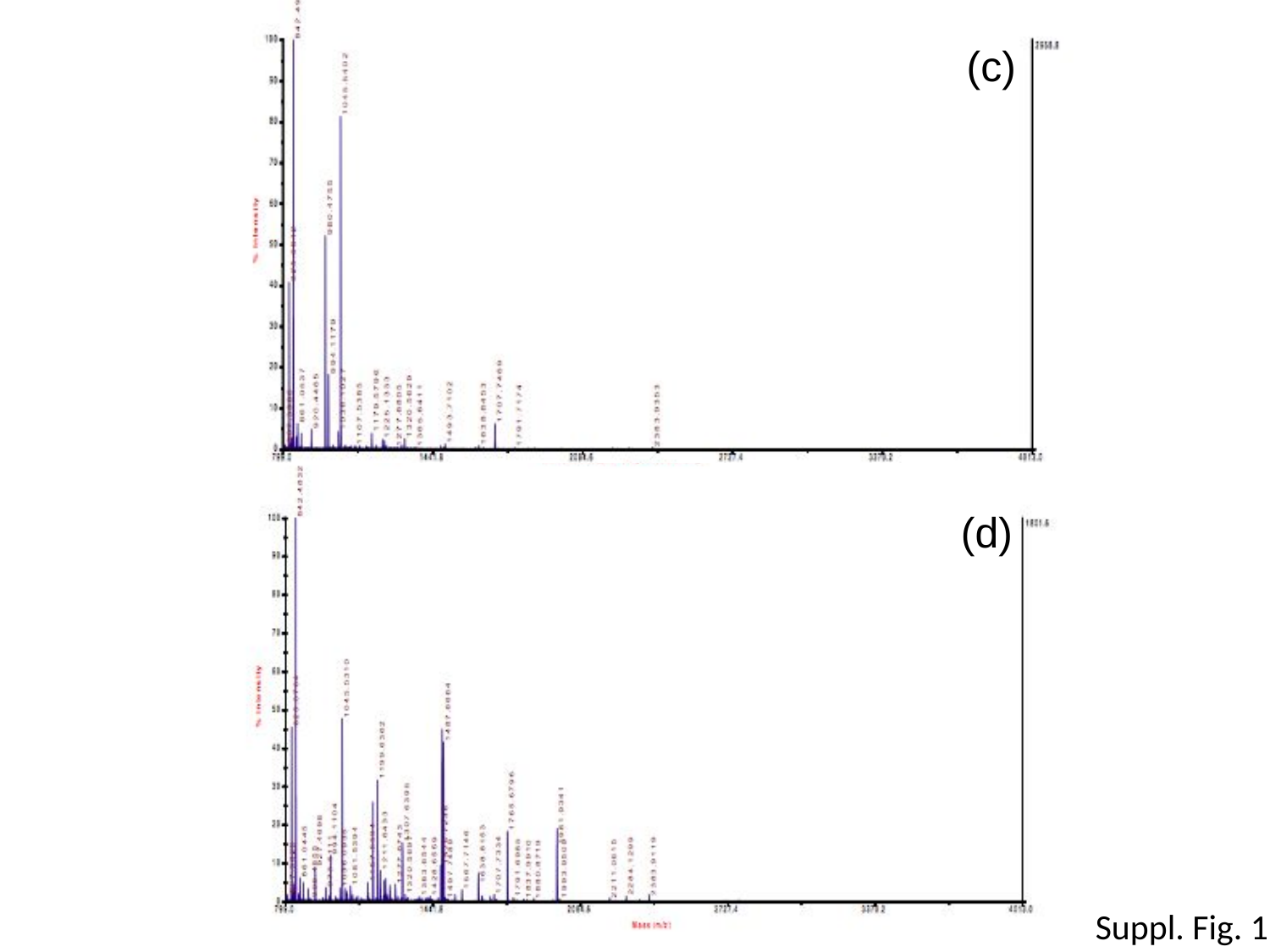

(c)
(d)
Suppl. Fig. 1

## Slide 4
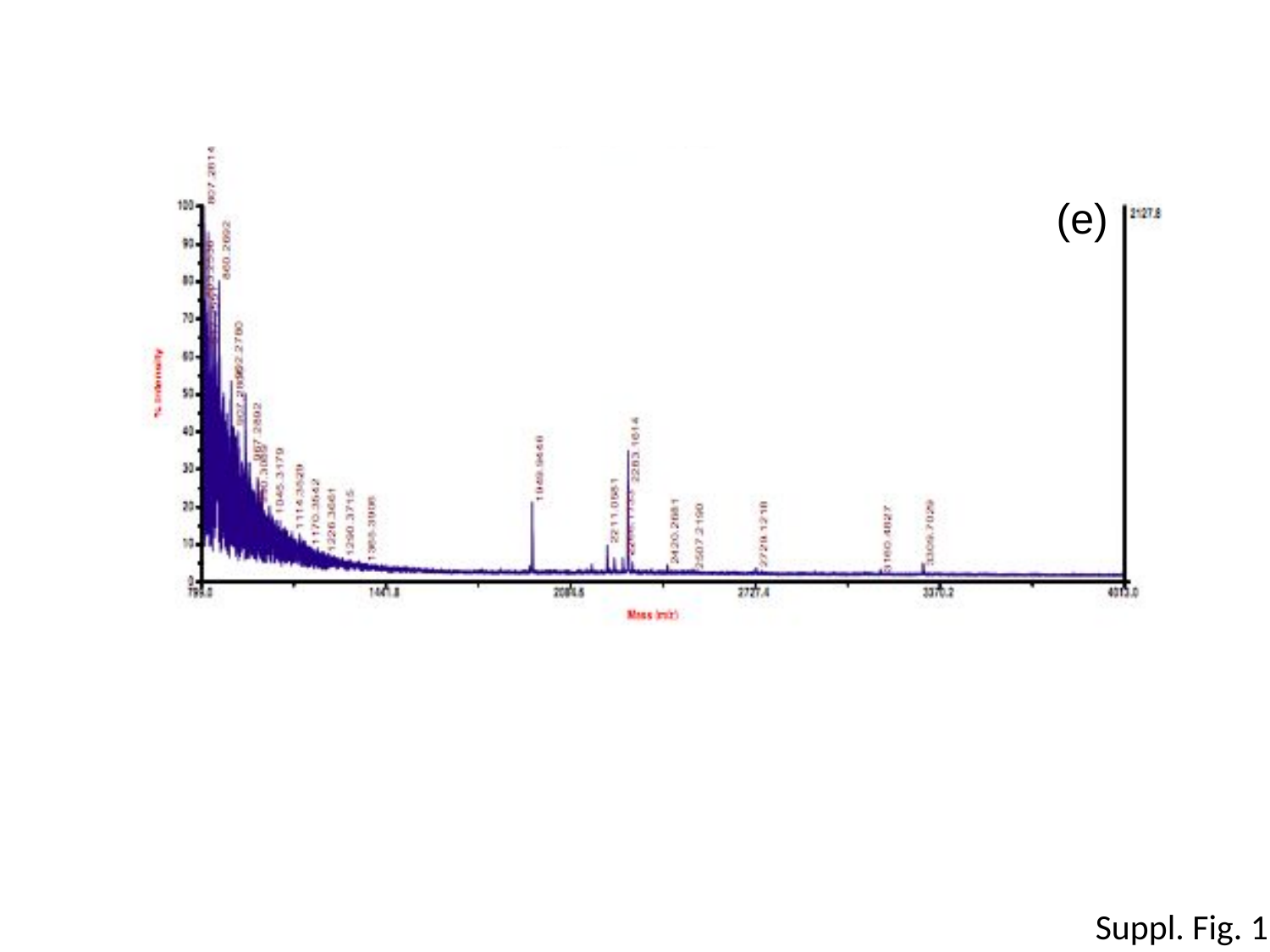

(e)
Suppl. Fig. 1

## Slide 5
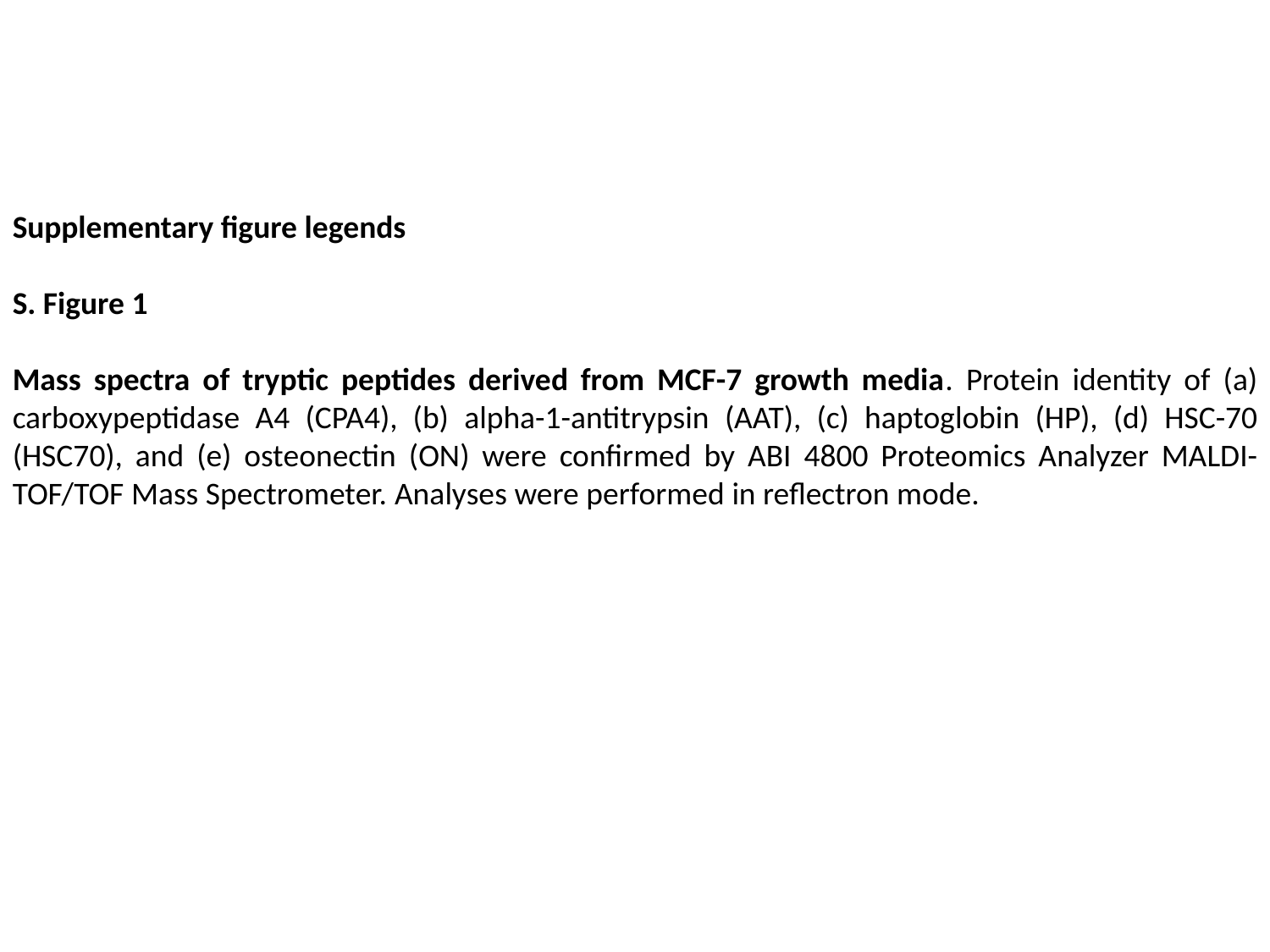

Supplementary figure legends
S. Figure 1
Mass spectra of tryptic peptides derived from MCF-7 growth media. Protein identity of (a) carboxypeptidase A4 (CPA4), (b) alpha-1-antitrypsin (AAT), (c) haptoglobin (HP), (d) HSC-70 (HSC70), and (e) osteonectin (ON) were confirmed by ABI 4800 Proteomics Analyzer MALDI-TOF/TOF Mass Spectrometer. Analyses were performed in reflectron mode.
